# A novel ACE2 decoy for both neutralization of SARS-CoV-2 variants and killing of infected cells

**DOI:** 10.3389/fimmu.2023.1204543

**Published:** 2023-06-13

**Authors:** Alexandra Kegler, Laura Drewitz, Claudia Arndt, Cansu Daglar, Liliana Rodrigues Loureiro, Nicola Mitwasi, Christin Neuber, Karla Elizabeth González Soto, Tabea Bartsch, Larysa Baraban, Holger Ziehr, Markus Heine, Annabel Nieter, Andres Moreira-Soto, Arne Kühne, Jan Felix Drexler, Barbara Seliger, Markus Laube, Domokos Máthé, Bernadett Pályi, Polett Hajdrik, László Forgách, Zoltán Kis, Krisztián Szigeti, Ralf Bergmann, Anja Feldmann, Michael Bachmann

**Affiliations:** ^1^ Department of Radioimmunology, Institute of Radiopharmaceutical Cancer Research, Helmholtz-Zentrum Dresden-Rossendorf (HZDR), Dresden, Germany; ^2^ Mildred Scheel Early Career Center, Faculty of Medicine Carl Gustav Carus, Technische Universität Dresden, Dresden, Germany; ^3^ Department of Pharmaceutical Biotechnology, Fraunhofer Institute for Toxicology and Experimental Medicine (ITEM), Braunschweig, Germany; ^4^ Institute of Virology, Charité-Universitätsmedizin Berlin, Corporate Member of Freie Universität Berlin, Humboldt-Universität zu Berlin, and Berlin Institute of Health, Berlin, Germany; ^5^ Medical Faculty, Martin-Luther-University Halle-Wittenberg, Halle, Germany; ^6^ Institute of Translational Immunology, Medical High School, Brandenburg an der Havel, Germany; ^7^ Department of Radiopharmaceutical and Chemical Biology, Institute of Radiopharmaceutical Cancer Research, Helmholtz-Zentrum Dresden-Rossendorf (HZDR), Dresden, Germany; ^8^ Department of Biophysics and Radiation Biology, Faculty of Medicine, Semmelweis University, Budapest, Hungary; ^9^ Hungarian Centre of Excellence for Molecular Medicine, In Vivo Imaging Advanced Core Facility, Szeged, Hungary; ^10^ CROmed Translational Research Ltd., Budapest, Hungary; ^11^ National Biosafety Laboratory, Division of Microbiological Reference Laboratories, National Public Health Center, Budapest, Hungary; ^12^ Semmelweis University School of Pharmacy, Semmelweis University, Budapest, Hungary; ^13^ National Center for Tumor Diseases Dresden (NCT), German Cancer Research Center (DKFZ), Faculty of Medicine and University Hospital Carl Gustav Carus, Technische Universität Dresden, Helmholtz-Zentrum Dresden-Rossendorf (HZDR), Dresden, Germany; ^14^ German Cancer Consortium (DKTK), Partner Site Dresden and German Cancer Research Center (DKFZ), Heidelberg, Germany

**Keywords:** SARS–CoV–2, COVID-19, ACE2 decoy, T-cell based immunotherapy, bispecific antibody, adapter CAR platform

## Abstract

The coronavirus disease 2019 (COVID-19) pandemic caused by severe acute respiratory syndrome coronavirus 2 (SARS-CoV-2) led to millions of infections and deaths worldwide. As this virus evolves rapidly, there is a high need for treatment options that can win the race against new emerging variants of concern. Here, we describe a novel immunotherapeutic drug based on the SARS-CoV-2 entry receptor ACE2 and provide experimental evidence that it cannot only be used for (i) neutralization of SARS-CoV-2 *in vitro* and in SARS-CoV-2-infected animal models but also for (ii) clearance of virus-infected cells. For the latter purpose, we equipped the ACE2 decoy with an epitope tag. Thereby, we converted it to an adapter molecule, which we successfully applied in the modular platforms UniMAB and UniCAR for retargeting of either unmodified or universal chimeric antigen receptor-modified immune effector cells. Our results pave the way for a clinical application of this novel ACE2 decoy, which will clearly improve COVID-19 treatment.

## 1 Introduction

At the beginning of 2020, severe acute respiratory syndrome coronavirus 2 (SARS-CoV-2) causing the serious, life-threatening coronavirus disease 2019 (COVID-19) started to spread worldwide, resulting in a pandemic with a devastating impact on human health and the global economy. Up until now, more than 765 million people got infected by the virus, leading to over 6.9 million fatal outcomes (1). Obviously, neither an infection nor repeated vaccination leads to long-lasting protection against new evolving SARS-CoV-2 variants (2–5). Thus, there is still a high risk of recurring infection outbreaks, as seen recently after China decided to relax its zero-COVID policy (6, 7). Moreover, the BQ and XBB subvariants, which are rapidly expanding at the moment, are resistant to all clinically authorized monoclonal antibodies (mAbs) (5). Consequently, new therapeutic options against currently existing and, most importantly, emerging future variants of SARS-CoV-2 are pressingly needed. Most desirable would be drugs that (i) are resistant to virus escape mechanisms, (ii) cannot only block infections (passive immunity), but (iii) may also be useful to recognize and destroy infected cells (active immunity). A molecule with these features could also become a prototype for the treatment of other infectious diseases.

In these regards, decoy molecules based on the respective viral entry receptor represent highly promising candidates: In the case of SARS-CoV-2, the viral entry site is the angiotensin-converting enzyme 2 (ACE2) receptor (8, 9). ACE2 decoys have already demonstrated high efficacy in blocking ancestry SARS-CoV-2 and all tested variants of concern (VOCs), including Alpha, Beta, Gamma, Delta, and Omicron, *in vitro* and *in vivo* (10–17).

Therefore, one attractive approach is to design ACE2-based decoys, which are not only able to block infection but also to clear infected cells in a steerable immune cell-dependent manner. This strategy might further improve the treatment of patients since an effective SARS-CoV-2-specific T-cell immunity correlates with an efficient resolution of COVID-19 (18, 19). It is commonly accepted that cytolytic T-cell activity is dependent on SARS-CoV-2 epitope presentation on major histocompatibility complex (MHC) class I molecules (20). However, like many viruses, SARS-CoV-2 downregulates the expression of MHC class I molecules and, thereby, impairs the MHC class I pathway of infected cells (21). Moreover, mutations in MHC class I-restricted epitopes and the expression of nonclassical HLA-G support SARS-CoV-2-mediated immune escape (22, 23). To overcome these obstacles, T-cell engaging bispecific Abs (bsAbs) or chimeric antigen receptor (CAR) modified T-cells with specificity for the receptor-binding domain (RBD) of SARS-CoV-2 could be applied, since SARS-CoV-2 infected cells exhibit the RBD containing Spike (S) protein on their surface (24) and both technologies enable MHC-independent antigen recognition (25, 26).

In general, bsAbs consist of two binding moieties with specificity for an antigen on target cells and an activating receptor on an effector cell. In the case of T-cells, bsAbs are directed to the CD3 complex and can cross-link target and effector cells, which leads to T-cell activation and the eradication of target cells (27). Similarly, T-cells modified to express a CAR, which typically possesses an extracellular target antigen-binding moiety, a transmembrane, and an intracellular T-cell activation domain, are able to specifically recognize and efficiently eliminate target cells (28). Both approaches have been successfully employed for the treatment of hematologic malignancies (29–31). On the downside, CAR-armed T-cells once applied in the human body cannot be rapidly and reversibly switched off and on, thereby bearing the risk of life-threatening adverse events such as off-target cell effects and cytokine storms (32).

Over the past two decades, we developed two modular platform technologies in order to overcome these hurdles. The modular bsAb platform was termed the UniMAB system (33) and the modular adapter CAR platform was named the UniCAR system (34, 35). Both technologies use immune complexes for the redirection of immune cells to target cells (32, 36). The functional immune complexes are formed between an immune cell activating component and target modules (TMs). The same TMs can be used for both the UniMAB and UniCAR platform technology. A TM contains an antigen-specific binding moiety and a small peptide epitope tag. In the case of the UniMAB system, the immune cell activating component is a bsAb, named effector module (EM), which recognizes on the one hand the peptide epitope tag of the TM, and on the other hand the CD3 complex of T-cells. As a result, the anti-CD3-anti-peptide bsAb can form an immune complex with the TM, which enables the redirection of unmodified T-cells to target antigen-expressing cells. In the case of the UniCAR system, the peptide epitope tag of the TM is recognized by the single-chain-fragment variable (scFv) of the UniCAR. Thus, T-cells genetically modified to express this artificial receptor can be cross-linked *via* the TM to target cells leading to UniCAR T-cell activation and target cell elimination. Importantly, in the absence of any TM, both platforms are inert. As a consequence, T-cell activity can be controlled by TM availability (32, 36). As another advantage, the UniCAR system also works when using natural killer (NK) cells or NK cell lines as effector cells instead of T-cells (37, 38). Both the UniMAB and the UniCAR technologies demonstrated great success in the *in vitro* and *in vivo* eradication of tumor cells from various origins (33, 39–48). Moreover, the UniCAR technology is currently being tested in two clinical phase I trials (NCT04230265, NCT04633148), in which already “proof of concept” for its safety feature, namely the repeated and reversible switching, could be confirmed and the first promising outcomes in treated patients were reported (49).

**Figure 1:**
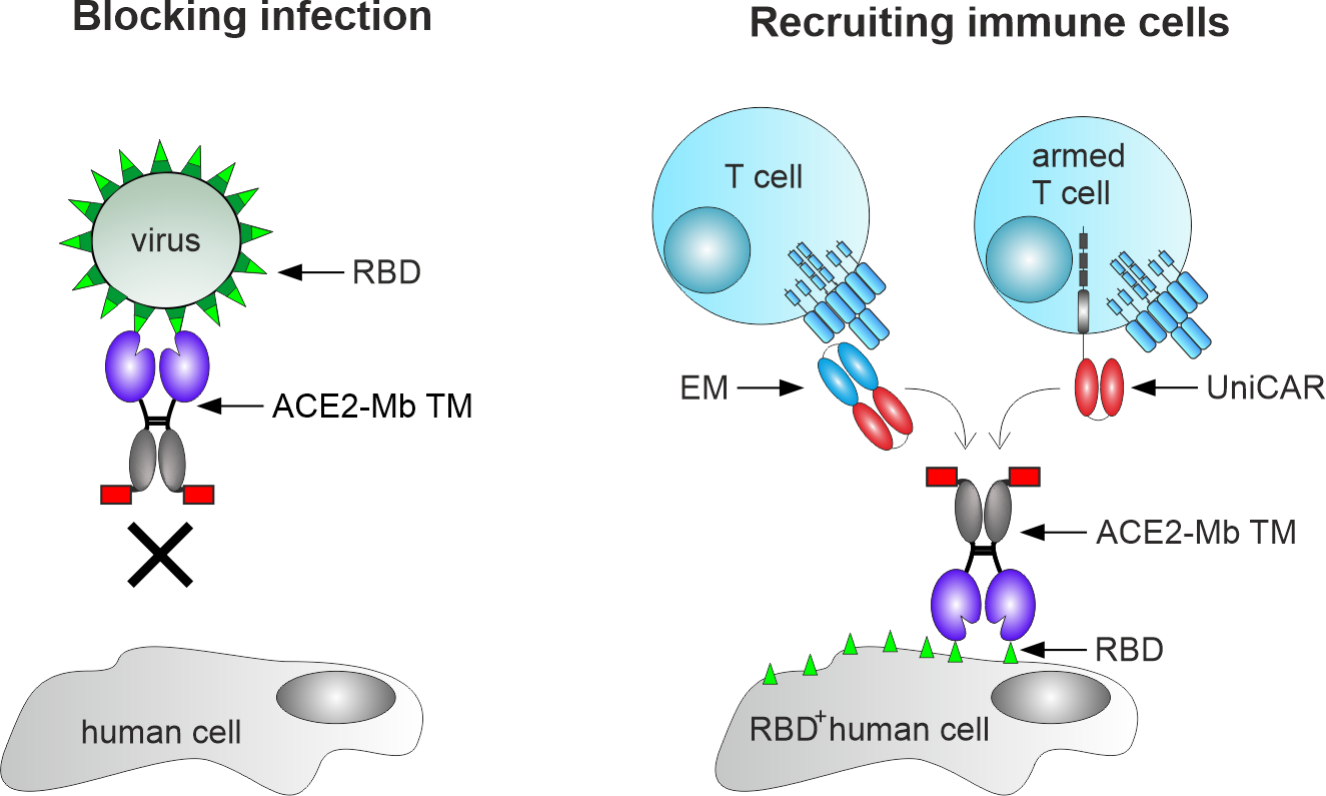
Mechanism of action of the angiotensin-converting enzyme 2 (ACE2)-minibody (Mb) target module (TM). Our novel TM is based on the extracellular domain of human ACE2 fused to the hinge and C_H_3 region of human IgG1, as well as the peptide epitope E5B9 (red box). Consequently, it should bind to the receptor-binding domain (RBD) of SARS-CoV-2 and block SARS-CoV-2 infections in human cells (left). In addition, this novel TM should be able to bind to virus-infected cells expressing SARS-CoV-2 RBD on their surface (RBD^+^ human cells), thereby mediating their elimination *via* T-cell-based immunotherapy (right). For this purpose, either unmodified T-cells in combination with the previously described effector module (EM) of the UniMAB system or T-cells equipped with the universal artificial receptor of the UniCAR system can be used for targeting virus-infected cells.

Having in mind (i) the capability of ACE2 decoy proteins to neutralize existing and most likely upcoming VOCs of SARS-CoV-2 and (ii) the high efficacy of the UniMAB and UniCAR systems to eliminate harmful cells in an MHC-independent manner, we decided to develop an ACE2-based decoy protein that cannot only block entry of SARS-related viruses but also be combined as a TM with the UniMAB and UniCAR systems to eliminate virus-infected cells (**Figure 1**).

## 2 Results

### 2.1 Construction, expression, and purification of the ACE2-based TM

**Figure 2:**
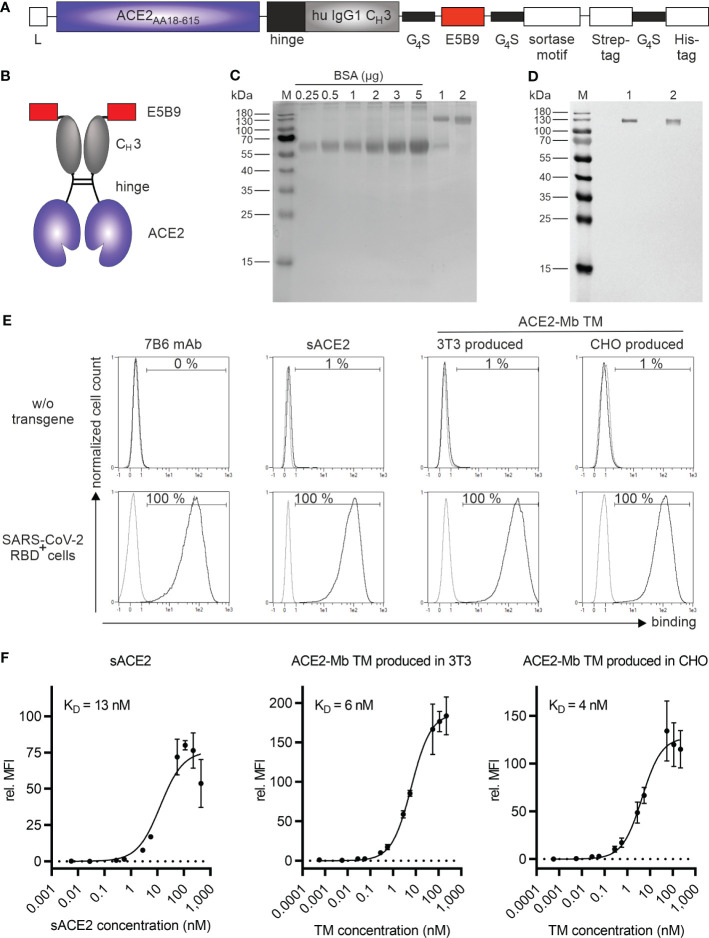
Biochemical and functional characterization of the ACE2-Mb TM. **(A)** Schematic representation of the ACE2-Mb TM containing a portion of the extracellular domain of the human ACE2 receptor (AA_18-615_) linked to the hinge and C_H_3 region of a human IgG1 Ab and the peptide tag E5B9. L, leader peptide. **(B)** As schematically shown, two ACE2-Mb TM monomers can form a homodimer *via* disulfide bonds in the hinge region. **(C, D)** The TM was recombinantly expressed in either (1) 3T3 or (2) a GMP-grade producer cell line derived from CHO cells. The ACE2-Mb TM was purified from cell culture supernatants *via* its Strep-tag. **(C)** In order to estimate the concentration and purity of the isolated ACE2-Mb TM, elution fractions were analyzed by SDS-PAGE followed by staining with Quick Coomassie^®^ Stain. **(D)** In order to identify the ACE2-Mb TM, SDS-PAGE and immunoblotting were performed. The TM was detected *via* its E5B9-tag. **(E, F)** Analysis of binding of the ACE2-Mb TM to the RBD domain of SARS-CoV-2 *via* flow cytometry. For this purpose, cell lines were established permanently expressing the SARS-CoV-2 RBD_WT_ fusion protein tagged with the E7B6 peptide epitope. **(E)** As a negative control, cells lacking the SARS-CoV-2 RBD_WT_ fusion protein (w/o transgene) were used. The binding of the ACE2-Mb TM was detected using as the primary Ab the anti-La mAb (5B9) and a fluorescently labeled secondary goat anti-mouse IgG (minimal x-reactivity) Ab. For anti-La mAb (7B6) and sACE2 detection, only fluorescently labeled goat anti-mouse IgG (minimal x-reactivity) Ab and anti-His mAb were applied, respectively. Histograms depict stained cells (black) and the respective control (grey). **(F)** To determine affinity towards SARS-CoV-2 RBD_WT_, cells were incubated with increasing amounts of sACE2, 3T3-, or CHO-produced ACE2-Mb TM. Relative median fluorescence intensity (rel. MFI) as mean ± SD and *K*_D_ values are shown (*n* = 3).

To develop the desired ACE2 decoy-based TM, we constructed an ACE2 Ig fusion molecule consisting of (i) the extracellular domain of the human ACE2 receptor (AA_18-615_), (ii) the hinge and C_H_3 region of a human IgG1 Ab (50), and (iii) the peptide epitope tag E5B9, against which the clinically used UniCAR version is also directed. The E5B9 epitope sequence is part of the nuclear autoantigen La/SS-B (32, 51). For analytical purposes and purification, we added a sortase motif, Strep- and His-tag downstream of the E5B9-tag (**Figure 2A**). Due to the incorporation of an IgG1 hinge, this novel ACE2-minibody (Mb) TM is able to dimerize *via* its disulfide bonds, resulting in homodimers with a theoretical molecular weight of 180 kDa (**Figure 2B**). Moreover, since the ACE2-Mb TM only contains, besides the hinge, the C_H_3 domain of an IgG1 Ab, it lacks the ability to interact with the FcγR and C1q. Thus, it should not activate FcγR- expressing immune cells and thereby antibody-dependent cellular cytotoxicity (ADCC) or the complement cascade.

After ACE2-Mb TM cloning, two producer cell lines secreting the molecule were established: In the first assays, TM expressed from a mouse 3T3 cell line was used. Later on, a production cell pool from a GMP-grade CHO cell line was established. The respective TMs were purified from cell culture supernatants using Strep-Tactin affinity chromatography. Isolated TMs were analyzed by SDS-PAGE under reducing conditions followed by immunoblotting. As shown in **Figures 2C, D**, a single band at approximately 130 kDa was obtained for both (1) 3T3 and (2) CHO-produced TM. The deviation from its theoretical size of 90 kDa may be due to posttranslational modifications.

### 2.2 Binding properties of the ACE2-Mb TM

In order to assess the binding of the ACE2-Mb TM to SARS-CoV-2 RBD, we constructed a vector encoding an artificial SARS-CoV-2 RBD_wild type (WT)_ membrane fusion protein. The extracellular RBD domain was tagged with the peptide epitope E7B6. The three cell lines HT-29 (colorectal carcinoma), NCI-H23 (lung carcinoma), and PC3 (prostate carcinoma) were transduced to express this artificial membrane protein. As shown in **Figure 2E**, the surface expression of the SARS-CoV-2 RBD_WT_ fusion protein was confirmed using a mAb directed against the E7B6-tag. Moreover, commercially available soluble ACE2 (sACE2) and both 3T3- and CHO-produced ACE2-Mb TM were able to specifically bind all SARS-CoV-2 RBD_WT_-expressing cells (**Figure 2E**). As depicted in **Figure 2F**, the novel ACE2-Mb TM had a high and, in comparison to the sACE2, enhanced binding affinity toward SARS-CoV-2 RBD_WT_. Furthermore, the binding affinity was comparable between TM expressed by 3T3 or CHO cells (*K*_D_ = 6 nM and *K*_D_ = 4 nM, respectively). Similar *K*_D_ values were obtained when using the transduced colorectal or lung cell lines (**Supplementary Figure S1**, *K*_D_ = 3 nM and *K*_D_ = 5 nM, respectively) instead of the genetically modified PC3 cell line (**Figures 2E, F**). Finally, we confirmed the binding capability of the ACE2-Mb TM to the SARS-CoV-2 RBD_WT_ domain using surface plasmon resonance (SPR) technology (*K*_D_ = 2 nM, see also **Supplementary Table S1**).

### 2.3 The ACE2-Mb TM blocks the binding of RBD to ACE2

**Figure 3:**
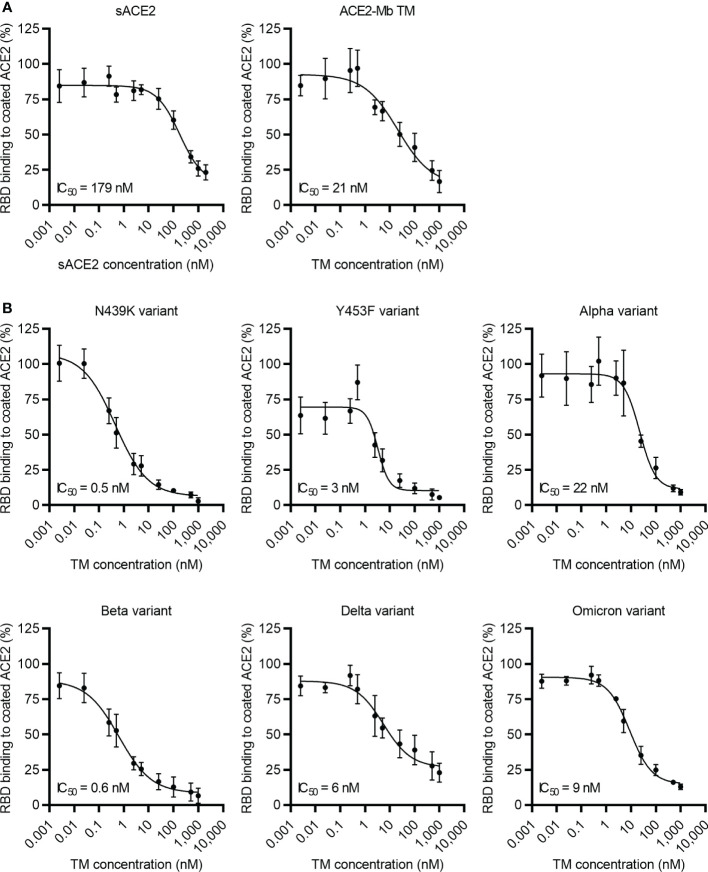
Blocking of ACE2 receptor binding. Increasing concentrations of **(A, B)** ACE2-Mb TM or **(A)** soluble ACE2 (sACE2) were added together with His-tagged **(A)** SARS-CoV-2 RBD_WT_ or **(B)** RBD from different SARS-CoV-2 variants on ELISA plates coated with human ACE2. The binding of RBD to coated ACE2 was detected by using an HRP-conjugated anti-His mAb. The data represent the mean ± SD and IC_50_ of three independent experiments.

After confirming the high affinity of the ACE2-Mb TM to SARS-CoV-2 RBD_WT_, its capacity to block the binding of this viral protein to coated ACE2 was evaluated in an ELISA-based assay. As shown in **Figure 3A**, the ACE2-Mb TM was able to efficiently inhibit this interaction. An IC_50_ value of 21 nM was estimated for the ACE2-Mb TM, which is superior compared to the approximately nine times lower IC_50_ value determined for the sACE2 (IC_50 _= 179 nM). Moreover, besides SARS-CoV-2 RBD_WT_, the ACE2-Mb TM potently blocked the binding of all tested SARS-CoV-2 RBD variants to coated ACE2 (**Figure 3B**). Its efficacy was clearly enhanced in the presence of nearly all SARS-CoV-2 RBD variants, as shown by a lower IC_50_ value.

### 2.4 Neutralization potential of the ACE2-Mb TM against SARS-CoV-2 S-pseudo-typed virus particles

**Figure 4:**
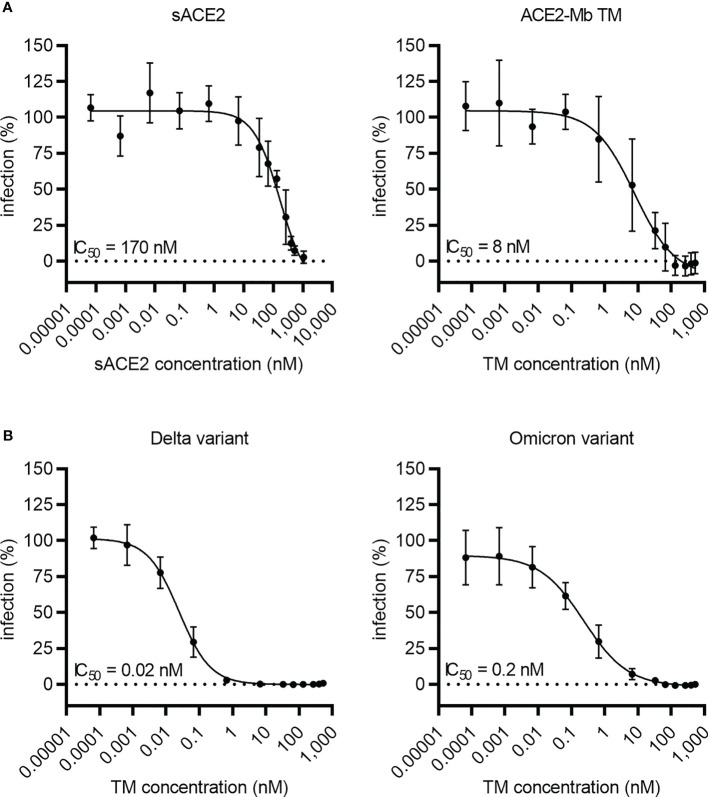
Blocking of infection by SARS-CoV-2 S-pseudo-typed virus particles. Luciferase-encoding virus particles pseudo-typed with **(A)** SARS-CoV-2 S_WT_ protein or **(B)** S protein from Delta and Omicron VOCs were added together with increasing concentrations of **(A, B)** ACE2-Mb TM or **(A)** soluble ACE2 (sACE2) to HEK 293T ACE2 cells. After 3 days, the infection rate was determined by using a bioluminescence-based assay. Summarized data are shown as mean ± SD and IC_50_ values are depicted (*n* ≥ 3).

To estimate the neutralization capability of the ACE2-Mb TM, a pseudo-virus assay was performed (**Figure 4**). In these experiments, the ACE2-Mb TM was able to efficiently prevent infection of HEK 293T ACE2 cells. In line with the ELISA data, the ACE2-Mb TM showed a higher neutralization efficacy against virus particles pseudo-typed with SARS-CoV-2 S_WT_ (IC_50 _= 8 nM) than sACE2 (IC_50 _= 170 nM) (**Figure 4A**). Importantly, in the presence of virus particles pseudo-typed with SARS-CoV-2 S protein from Delta and Omicron VOCs, the neutralization capacity of the ACE2-Mb TM was even more pronounced (**Figure 4B**). The obtained IC_50_ values were 400 and 40 times lower, respectively, when compared to SARS-CoV-2 S_WT_ harboring virus particles were added. These data are in accordance with both SPR binding (RBD_WT_: *K*_D_ = 2 nM, RBD_Delta_: *K*_D_ = 0.6 nM, RBD_Omicron_: *K*_D_ = 0.5 nM, **Supplementary Table S1**) as well as ELISA results (RBD_WT_: IC_50 _= 21 nM, RBD_Delta_: IC_50 _= 6 nM, RBD_Omicron_: IC_50 _= 9 nM; **Figure 3**) and indicate that the ACE2-Mb TM is a very potent agent for neutralizing SARS-CoV-2, including VOCs.

### 2.5 The ACE2-Mb TM efficiently inhibits SARS-CoV-2 and VOC infectivity

**Figure 5:**
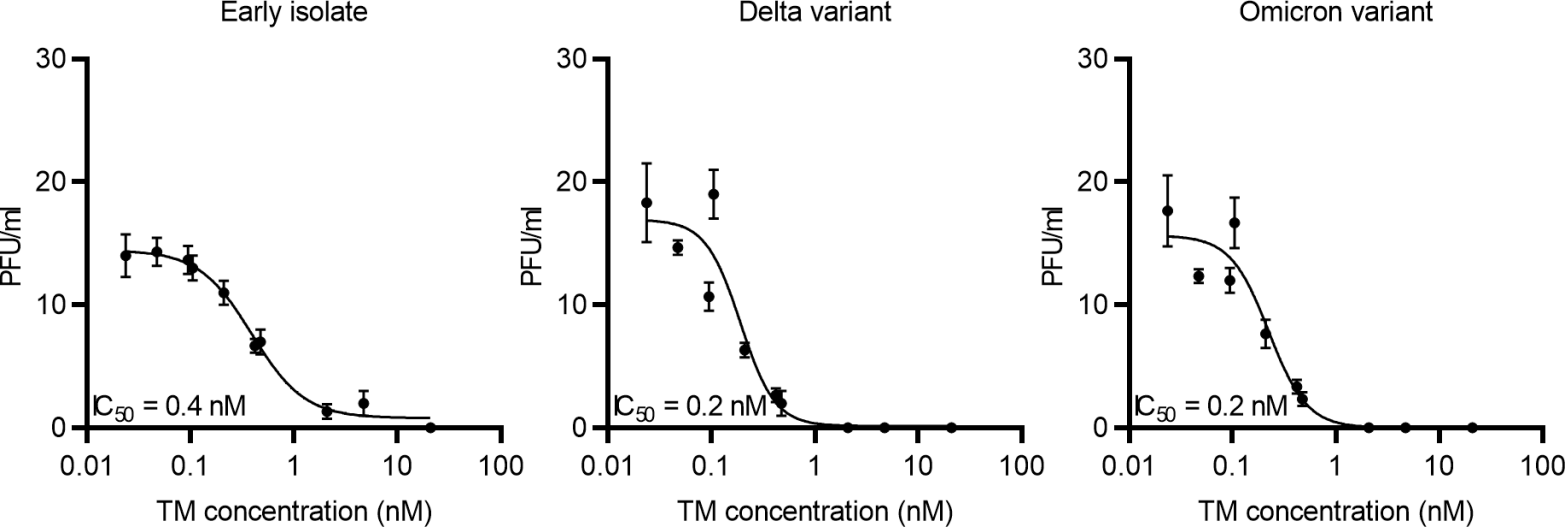
Blocking of infection of live SARS-CoV-2. Increasing concentrations of the ACE2-Mb TM were incubated with an early isolate, Delta or Omicron VOC, for 1 h at 37°C and subsequently added to Vero E6 cells. After 1 h, Avicel/DMEM was supplemented, and plates were incubated for 2 to 3 days followed by fixation, inactivation, crystal violet staining, and plaque number counting. Plaque-forming units (PFU) per milliliter were plotted against the TM concentration. Summarized data of triplicates as mean ± SD and IC_50_ values are shown.

Since the ACE2-Mb TM efficiently blocks RBD binding to ACE2 (**Figure 3**) and S-pseudo-typed virus particle entry into human host cells (**Figure 4**), its capability to inhibit live SARS-CoV-2 infection was determined using a plaque reduction neutralization assay (PRNT). As depicted in **Figure 5**, the ACE2-Mb TM efficiently neutralized an early isolate of the virus as well as the Delta and Omicron VOCs with IC_50_ values of 0.4 nM (early isolate) and 0.2 nM (Delta and Omicron VOCs), demonstrating that the ACE2-Mb TM possesses an antiviral effect even at picomolar concentrations. These data further emphasize the high therapeutic potential of the ACE2-Mb TM as an effective neutralization agent.

### 2.6 The ACE2-Mb TM efficiently redirects T-cells to kill SARS-CoV-2 RBD^+^ target cells

**Figure 6:**
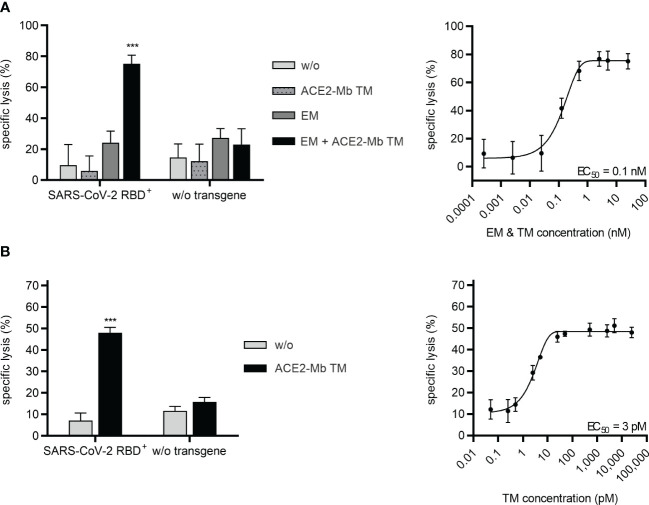
Recruitment of T-cells *via* the UniMAB or UniCAR system results in efficient lysis of SARS-CoV-2 RBD^+^ cells in the presence of the ACE2-Mb TM. **(A, B)** Bioluminescence-based cytotoxicity assays were performed with luciferase^+^ PC3 cells either possessing (SARS-CoV-2 RBD^+^, left and right diagrams) or lacking (w/o transgene, left diagrams) SARS-CoV-2 RBD expression. These PC3 cells were coincubated at an *E*:*T* ratio of 5:1 with **(A)** unmodified T-cells for 24 h or **(B)** UniCAR T-cells for 6 h in the absence (w/o) or presence of 25 nM (left diagrams) or decreasing concentrations (right diagrams) of indicated Ab components. Summarized data of three individual donors as mean ± SD and EC_50_ values are shown. Statistical analysis was performed by using two-way ANOVA with **(A)** Tukey’s or **(B)** Šidák’s multiple comparisons tests (^***^ *p* < 0.001 with respect to **(A, B)** w/o and **(A)** only ACE2-Mb TM as well as only EM controls).

Next, it was investigated whether the ACE2-Mb TM not only efficiently neutralizes SARS-CoV-2, including Delta and Omicron VOCs, but also induces T-cell-mediated immune responses against virus-infected cells. PC3 cells expressing the SARS-CoV-2 RBD_WT_ fusion protein were used as surrogate model. As shown in **Figure 6A**, when the ACE2-Mb TM was applied in the presence of T-cells and the EM of the UniMAB system, a high specific lysis of SARS-CoV-2 RBD^+^ cells was observed, whereas neither the ACE2-Mb TM nor the EM alone triggered T-cells to kill target cells. Moreover, no increased lysis could be detected when cells lacking SARS-CoV-2 RBD on their surface were applied. Thus, target cell eradication is strictly dependent on the TM/EM-mediated cross-linkage between T-cells and SARS-CoV-2 RBD^+^ target cells, underlining the capability and high specificity of redirecting unmodified T-cells with the combination of the ACE2-Mb TM and the EM of the UniMAB system. By titrating both Ab components, an EC_50_ value of 0.1 nM was determined, showing that this UniMAB application is extremely efficient even at picomolar concentrations (**Figure 6A**).

Similar results were obtained when the EM of the UniMAB system was replaced by T-cells genetically modified to express UniCARs. As shown in **Figure 6B**, UniCAR T-cells were able to efficiently kill SARS-CoV-2 RBD^+^ target cells, which was solely observed in the presence of the ACE2-Mb TM. Based on titration experiments, an EC_50_ value of 3 pM was determined for the UniCAR approach, demonstrating potent lysis of target cells by UniCAR T-cells at low picomolar TM concentrations (**Figure 6B**).

As expected, cytokine secretion was only triggered after cross-linkage of unmodified T-cells *via* the EM/TM-immune complex (**Supplementary Figure S2**) or of UniCAR T-cells *via* the TM (**Supplementary Figure S3**) to SARS-CoV-2 RBD^+^ cells.

Taken together, high specificity and efficiency were proven for both technologies, underlining the great potential of the UniMAB and UniCAR systems as powerful tools to eradicate SARS-CoV-2- infected cells.

### 2.7 Nasal application of the ACE2-Mb TM efficiently inhibits the infection of experimental hamsters with SARS-CoV-2

**Figure 7:**
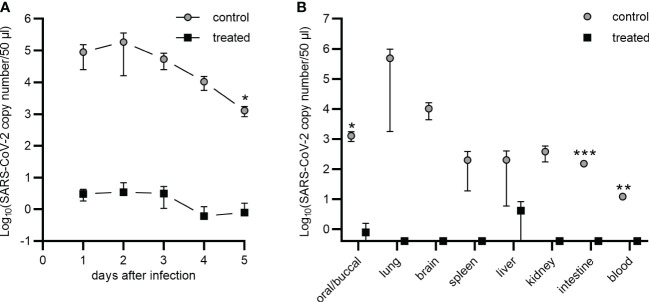
Nasal application of the ACE2-Mb TM protects Syrian hamsters against SARS-CoV-2 infection. **(A, B)** Five Syrian hamsters were infected by intranasal inoculation of the SARS-CoV-2 Delta VOC (B.1.617.2) in the absence (control) or presence (treated) of the ACE2-Mb TM (see Material and methods). Virus load was estimated by qRT-PCR in **(A)** oral/buccal samples collected over 5 days after infection or **(B)** in oral/buccal samples, blood, and depicted organs harvested at day 5 after infection. **(A, B)** Summarized data of two (control) or three (treated) animals are shown as mean ± SEM (data points on the *x*-axis represent the value 0). Statistical analysis was performed by using an unpaired two-tailed Student’s *t*-test (^*^ *p* < 0.05; ^**^ *p* < 0.01; ^***^ *p* < 0.001).

In order to further corroborate the blocking capability of the ACE2-Mb TM *in vivo* and to test whether or not a local (intranasal) administration could protect against infection, Syrian hamsters were infected with the SARS-CoV-2 Delta VOC either in the presence (**Figure 7**, treated) or absence (**Figure 7**, control) of the ACE2-Mb TM. For the treatment group, SARS-CoV-2 in VP-SFM was mixed with the ACE2-Mb TM in PBS, whereas for the control group, the same virus suspension was solely mixed with PBS in equal parts. The hamsters were infected by intranasal application of 80 µl of the respective virus-containing suspension. In the following days, the viral load of oral/buccal samples was estimated by RT-PCR (**Figure 7A**), and animal body weight was measured (**Supplementary Figure S4**). Animals were sacrificed 5 days after infection, and the viral load in the organs, including the lung, brain, spleen, liver, kidney, intestine, and blood was determined (**Figure 7B**). As shown in **Figures 7A, B**, the intranasal application of the ACE2-Mb TM efficiently blocked the infection and protected all organs analyzed. In this experimental setup, the therapeutic efficacy of the ACE2-Mb TM should be solely based on its neutralization properties since (i) it lacks the ability to induce ADCC or the complement cascade and (ii) neither UniCAR T-cells nor EM of the UniMAB system were co-injected.

## 3 Discussion

As SARS-CoV-2 is a rapidly evolving virus with the fast emergence and spread of various sublineages (52, 53), the high occurrence of novel mutations represents a major challenge for the development of vaccines and mAb therapies (2, 3, 54–56). By contrast, ACE2 decoys show high neutralization efficacy against SARS-CoV-2, including all so far known VOCs (10–17). Besides SARS-CoV-2, ACE2 decoys were shown to efficiently block the entry of other sarbecoviruses, such as SARS-CoV-1, Pangolin CoV GD-1, and bat CoV RsSHC014, into human cells (12). Interestingly, when SARS-CoV-2 was passaged in Vero E6 cells in the presence of an ACE2 decoy, no escape mutations were observed in the RBD (17), whereas multiple amino acid substitutions occurred in the RBD under the selection pressure of single mAbs in a similar assay (57). Thus, in contrast to mAbs, ACE2 decoys seem to be resistant to SARS-CoV-2 mutations and might not foster their emergence under treatment conditions. In addition, ACE2 decoys diminish S protein-mediated syncytia formation between adjacent cells (17), which is considered to be a pathological feature of COVID-19 with pulmonary manifestation (58). Due to all these advantages, ACE2 decoys seem to be the drug format of choice to protect cells against infection by blocking the entry of SARS-CoV-2 and future emerging variants.

T-cell-mediated cellular immunity could further improve the defense against infection *via* the clearance of virus-infected cells. However, viruses impair the recognition mechanism of effector T-cells, e.g., by downregulating MHC class I molecules (21). Bearing in mind that SARS-CoV-2-infected cells reveal themselves through the presence of viral proteins on their cell surface (24), we decided to convert an ACE2 decoy into a TM for targeting infected cells with either unmodified T-cells using the UniMAB system or genetically modified UniCAR T-cells.

During the construction of such an ACE2-based TM, a series of decisions had to be made: (i) ACE2 is an enzyme. Interestingly, it has been shown that the proteolytic activity of ACE2 is beneficial in acute lung injury animal models (59) and might, therefore, also be protective against COVID-19. Recently, this assumption was confirmed in mice infected with SARS-CoV-2, underlining the dual mode of action of catalytically functional ACE2 decoys as virus-neutralizing and anti-inflammatory drugs (60). Moreover, the tolerability of an enzymatically active ACE2-based therapeutic agent was already demonstrated in healthy subjects (61) and patients with acute respiratory distress syndrome (62), pulmonary arterial hypertension (63), or COVID-19 (64). For these reasons, the here-described ACE2-Mb TM contains an extracellular portion of ACE2 without mutation in the catalytic site. In the case of a clinical application, we therefore expect that, besides its antiviral functions, the ACE2-Mb TM should be safe and may even have anti-inflammatory properties.

(ii) Obviously, the novel ACE2-based TM must have a sufficiently high affinity and suitable pharmacological features. From our previous studies, we know that TMs with a low molecular weight are rapidly eliminated (39) and that their half-life is, therefore, presumably too short for an efficient, long-lasting blocking of virus entry. Fusion of target binding domains to an Ig-backbone not only extends the half-life of the TM but also increases the number of binding sites and, thereby, the apparent binding affinity (65). Unfortunately, Ab-based molecules can deliver viruses to FcγR expressing cells and, thereby, promote infection, leading to an increase in the virus load, a process known as antibody-dependent enhancement (ADE). Moreover, they can foster inflammation *via* the activation of FcγR-expressing immune cells and the complement cascade (66). For the construction of the ACE2-based TM, we therefore went for a Mb backbone consisting of the hinge and C_H_3 domain of an IgG1 Ab. As the resulting ACE2-Mb TM lacks those amino acids needed for interaction with the FcγR and C1q of the complement cascade (67), we expect that the ACE2-Mb TM should not harbor the risk of COVID-19 exacerbation *via* ADE. Due to the presence of the hinge region of an IgG1 Ab, the ACE2-Mb TM is able to form homodimers with two binding moieties. This concept led to the high affinity of the ACE2-Mb TM towards RBD being in the low nanomolar range of natural Abs evoked by SARS-CoV-2 infection (68). This high-affinity binding resulted not only in a potent blocking of the RBD/ACE2 interaction but also in an efficient neutralization of both pseudo-typed and live SARS-CoV-2. Moreover, the ACE2-Mb TM efficiently protected hamsters against an intranasal infection with SARS-CoV-2 (Delta variant). Interestingly, the ACE2-Mb TM showed stronger binding, blocking, and neutralization efficacy when SARS-CoV-2 VOCs or components thereof were applied in the respective experimental setup. Even in the presence of virus particles pseudo-typed with S proteins from the currently circulating subvariants, BQ.1.1 and XBB.1.5, highly efficient neutralization was observed (data not shown), which corroborates the hypothesis that the ACE2-Mb TM is resistant to virus escape mechanisms. These data are in line with the literature demonstrating that SARS-CoV-2 RBD of various variants possess a clearly increased binding affinity to ACE2 in comparison to RBD from the ancestral strain (69). Thus, it can be speculated that the ACE2-Mb TM may also show enhanced neutralization in patients infected with current or future emerging SARS-CoV-2 variants and thereby lead to an even improved treatment efficacy.

Finally, we tested the use of the ACE2-Mb TM for retargeting T-cells to RBD-expressing cells, thus simulating virus-infected cells. Indeed, when the ACE2-Mb TM was applied in combination with the EM of the UniMAB system, unmodified T-cells efficiently eliminated RBD^+^ human cells in an MHC-independent manner. In addition, we showed that the ACE2-Mb TM can mediate the efficient and selective killing of RBD^+^ human cells by UniCAR T-cells.

Based on these results, we expect that both the UniMAB and UniCAR systems should also be able to evoke a profound T-cell response in patients against virus-infected cells and, thereby, efficiently eliminate the viral reservoir. Thus, an early application of the ACE2-Mb TM in combination with the EM of the UniMAB system would be favorable, especially for patients with a high risk of severe COVID-19, as it would not only exert its virus-neutralizing and anti-inflammatory functions but also redirect the patient´s T-cells to kill virus-infected cells by forming a complex with the EM. Especially in immunocompromised COVID-19 patients with low numbers of T-cells, the UniCAR technology may become an alternative possibility. During the pandemic, a severely increased COVID-19-related mortality was, for instance, seen in immunocompromised recipients of conventional CAR T-cell immunotherapy (70, 71). In that regard, the modular UniCAR platform technology represents a promising solution, as it would kill two birds with one stone. Cancer patients, who were already treated with UniCAR T-cells and a tumor antigen-specific TM, would only need to receive the ACE2-Mb TM in addition when severely suffering from COVID-19. As a consequence, the ACE2-Mb TM would not only function as a virus-neutralizing and anti-inflammatory drug but also redirect UniCAR T-cells originally administered to the patient as a cancer treatment to now also eliminate virus-infected cells. In cases where autologous UniCAR T-cells are not available, the infusion of allogeneic UniCAR T- or UniCAR NK-cells together with the ACE2-Mb TM might also be an effective therapeutic option.

In conclusion, the presented work paves the way for a clinical application of the ACE2-Mb TM, which is both a highly promising protective and therapeutic agent, including for the treatment of immunocompromised patients and/or patients with a high risk of developing a severe COVID-19 disease. In addition, the data provide the first “proof of concept” for the use of the UniMAB and UniCAR platforms not only for targeting tumor cells but also for killing virus-infected cells. The recurrence of latent viruses, e.g., after bone marrow transplantation or CAR T-cell therapy, is a major concern in immunocompromised patients (72, 73). As adapter CAR technologies, such as the UniCAR system, allow the simultaneous application of different TMs, the recurrence of latent virus infections may be treated in parallel to tumor therapy following a similar concept as described here for SARS-CoV-2 infection.

## 4 Material and methods

### 4.1 Cell lines

The PC3-PSCA PSMA Luc, HT-29 Luc, and NCI-H23 Luc cell lines were genetically modified using a lentiviral system as previously described (74, 75) to further express a transgene encoding an N-terminal Igκ leader sequence, RBD_WT_ (AA_319-541_ of SARS-CoV-2 S protein), an E7B6-tag, as well as a human CD28-derived hinge and transmembrane region (SARS-CoV-2 RBD_WT_ fusion protein), followed by a 2A protease “cleavage” site and EGFP. PC3 and HT-29 cells were cultured in RPMI complete medium (35), whereas NCI-H23, HEK 293T, 3T3, and Vero E6 cells were kept in DMEM complete medium (35), unless otherwise stated. All these cells were kept at 37°C and 5% CO_2_ in a humidified atmosphere.

CHO cells were cultured in ActiPro medium supplemented with 8 mM L-glutamine as well as 1 µM MTX and subcultivated every 3 to 4 days (inoculation cell density: 3 × 10^5^ cells/ml). The cells were kept in shake flasks in a humid atmosphere at 37°C and 8% CO_2_ under orbital shaking (50 mm orbit) with 125 rpm.

### 4.2 Construction and expression of the ACE2-Mb TM

The human ACE2 sequence from AA_18-615_ was synthesized by Eurofins Genomics GmbH (Ebersberg, Germany). This sequence was subsequently inserted into the lentiviral vector p6NST50, already containing an N-terminal Igκ leader sequence and a C-terminal hinge and C_H_3 region of a human IgG1 Ab, E5B9-tag, sortase motif, Strep- and His-tag *via* the restriction enzymes *Sfi*I/*Mre*I (Thermo Fisher Scientific, Hennigsdorf, Germany). The E5B9-tag is part of the primary amino acid sequence of the nuclear autoantigen La/SS-B (51, 76). It is a continuous peptide sequence that is cryptic in native human La protein (32). Until now, no immunogenicity has been found, including in autoimmune patients developing anti-La autoantibodies (77). The resulting open reading frame (orf) encoding the ACE2-Mb TM was transduced into 3T3 cells using a lentiviral system as described previously (74, 75). For recombinant Ab expression, 2 × 10^6^ 3T3 cells were seeded in T175 cell culture flasks, and supernatants were harvested after 96 h.

Additionally, the ACE2-Mb TM-encoding orf was cloned into Fraunhofer’s own vector pHIT-basisDHFR2.0. The resulting vector pHIT-basisDHFR_Immunovid19 was transfected into Fraunhofer’s own CHO host cell line CHO-HITB7 by electroporation (AMAXA^®^ Nucleofector^®^ system, Lonza Group AG, Basel, Switzerland) after amplification and sequence verification. Subsequently, the selection of ACE2-Mb TM-producing CHO cells was performed by culturing with ProCHO™ 5 cultivation medium without hypoxanthine and thymidine (Lonza Group AG). After the selection phase, the expression was increased by adding 250 nM MTX and regularly checked. A research cell bank was deposited with the selected cells. For the production of the ACE2-Mb TM, cultivation was performed in a fed-batch mode in a shake flask scale for 14 days. ActiPro (powder), 8 mM L-glutamine, and an anti-clumping agent (1:100) were used as the basic medium. Every 2 days from day 3, 1% (v/v) HyClone™ CellBoost 7a supplement and 0.1% (v/v) HyClone™ CellBoost 7b supplement (both purchased from Cytiva, Marlborough, MA, USA) were added to the culture. The glucose concentration was maintained at 5 g/L throughout the production by adding a 40% (w/w) glucose stock solution. Depth filtration with the filter 60SP02A (3M) was performed to separate the cells after cultivation. Productivity was estimated by Bradford assay after purification *via* the Strep-tag. The productivity was 250 mg/L.

The generation of the 3T3 cell line expressing the EM of the UniMAB system was previously described (78).

### 4.3 Purification of the ACE2-Mb TM

The ACE2-Mb TM was purified from cell culture supernatant *via* its Strep-tag using a Strep-Tactin^®^XT buffer set and respective columns (IBA-Lifesciences, Göttingen, Germany) according to the manufacturer´s instructions. For EM purification *via* its His-tag, cell culture supernatant was added to PBS-equilibrated Ni-NTA agarose (Qiagen, Hilden, Germany) in Poly-Prep^®^ chromatography columns (Bio-Rad Laboratories GmbH, Feldkirchen, Germany). After two consecutive washing steps with 1 × PBS containing 150 mM NaCl and 10 or 20 mM imidazole, respectively, the EM was eluted using 1 × PBS with 150 mM NaCl and 350 mM imidazole. Isolated Ab constructs were dialyzed over night against 1 × PBS at 4°C and analyzed *via* SDS-PAGE followed by staining with Quick Coomassie^®^ Stain (Serva Electrophoresis GmbH, Heidelberg, Germany) or immunoblotting. In the latter case, a Trans-Blot Turbo RTA Mini 0.2 µm Nitrocellulose Transfer Kit (Bio-Rad Laboratories GmbH) and a DIG Wash and Block Buffer Set (Roche Diagnostics Deutschland GmbH, Mannheim, Germany) were used according to the manufacturer’s instructions. For TM detection, the anti-La mAb (5B9) (79) and an AP-conjugated rabbit-anti-mouse IgG Ab (Dianova GmbH, Hamburg, Germany) were applied.

### 4.4 Flow cytometric analysis

To analyze SARS-CoV-2 RBD fusion protein surface expression, cells were stained with 0.5 µg of an anti-La mAb (7B6) (80) or His-tagged sACE2 (0.5 µg or increasing amounts, trenzyme GmbH, Konstanz, Germany) for 1 h at 4°C, followed by 30 min of incubation at 4°C with a fluorescently labeled goat anti-mouse IgG (minimal x-reactivity) Ab (BioLegend, San Diego, CA, USA) or a fluorescently labeled anti-His mAb (Miltenyi Biotec GmbH, Bergisch Gladbach, Germany), respectively.

For analysis of TM binding, cells were incubated with 0.5 µg or increasing amounts of the ACE2-Mb TM for 1 h at 4°C. Afterwards, TM binding was detected by consecutive staining with 0.25 µg of the anti-La mAb (5B9) and the fluorescently labeled goat anti-mouse IgG (minimal x-reactivity) Ab (BioLegend). Cells were analyzed using a MACSQuant^®^ Analyzer 10 and MACSQuantify^®^ software (Miltenyi Biotec GmbH). *K*_D_ values were calculated with GraphPad Prism 9 software (GraphPad Software Inc., La Jolla, CA, USA).

### 4.5 Surface plasmon resonance

The SPR analyses were carried out on a Biacore T200 (GE Healthcare, Chicago, IL, USA) at 25°C using CM5 sensor chips (Cytiva) and HBS-P+ as running buffer (Cytiva) at a flow rate of 30 µl/min unless otherwise specified and a data collection rate of 10 Hz. All flow cells (FC) were normalized, and functionalization was performed on two consecutive FC using the anti-La mAb (5B9) and an amine coupling kit (GE Healthcare). The amine coupling procedure comprises the surface activation by EDC/NHS, the coupling step using a solution of the anti-La mAb (5B9) (10 µg/ml in 10 mM acetate buffer (pH 5.5) for 500 s at a flow of 10 µl/min), and the blocking of the surface using 1 M ethanolamine (pH 8.5). By that, 4130 RU (FC(reference)) and 5675 RU (FC(active)) of the anti-La mAb (5B9) was immobilized on the sensor surface.

Binding analyses were performed by a capturing approach with the ACE2-Mb TM as the ligand on FC(active), followed by a single-cycle-kinetic on both FC. Two start-up cycles were carried out at the beginning of each experimental set by capturing the ACE2-Mb TM (5 µg/ml in HBS-P^+^; 180 s; flow rate 10 µl/min) on FC(active), followed by three regeneration cycles using 3 M MgCl_2_ (diluted from 4 M MgCl_2_ (Cytiva) with water; each cycle 120 s at a flow of 10 µl/min) on both FC, and a final stabilization time of 300 s. For analyses, the ACE2-Mb TM (5 µg/ml in HBS-P+) was captured for 180 s at a flow rate of 10 µl/min on FC(active) with a stabilization time of 90 s, followed by a consecutive binding analysis of five increasing concentrations (single-cycle-kinetics) of the respective RBD variants (120 s contact time for each concentration followed by 600 s dissociation time; flow rate of 30 µl/min) on both FC. The regeneration of both FC was then performed in three regeneration cycles as described above. Blank runs using only buffer (HBS-P+) instead of the sample were carried out at the beginning and always after three cycles with the analyte to obtain double-referenced chromatograms.

Binding analyses were performed for each given concentration range in triplicate using the following analytes: RBD_WT_ (trenzyme GmbH, 1.85–150 nM in threefold dilution and 0.24–150 nM in fivefold dilution), RBD_Delta_ (trenzyme GmbH, 0.06–40 nM in fivefold dilution and 0.08–20 nM in fourfold dilution) and RBD_Omicron_ (GenScript Biotech Corporation, Piscataway, NJ, USA, 0.07–41.5 nM in fivefold dilution and 0.08–20 nM in fourfold dilution). Data were analyzed using the Biacore Evaluation software 3.0. Each sensorgram was reference subtracted (FC(active)-FC(reference)) and blank corrected with the mean of all blank runs (using buffer instead of the ligand as described above). Data were fitted to a 1:1 binding model.

### 4.6 ELISA-based binding inhibition assay

ELISA-based binding inhibition assays were performed using BD OptEIA™ Reagent Set B (BD Biosciences, Heidelberg, Germany) according to the manufacturer’s instructions. In brief, 96-well plates were coated with 9 nM ACE2 (trenzyme GmbH) per well overnight at 4°C. After blocking, His-tagged RBD (10 nM/well, purchased from trenzyme GmbH or Genscript Biotech Corporation) was added alone or together with increasing concentrations of the ACE2-Mb TM or sACE2 and incubated for 2 h at RT. His-tagged RBD bound to coated ACE2 was detected using an HRP-conjugated anti-His mAb (Miltenyi Biotec GmbH). HRP activity was, subsequently, measured at 450 nm using the GloMax^®^ Explorer System GM3500 (Promega GmbH, Walldorf, Germany). RBD binding to coated ACE2 in the absence of the ACE2-Mb TM or sACE2 (MAX) was equalized to 100% and relative RBD binding to coated ACE2 in the presence of the ACE2-Mb TM or sACE2 was calculated with respect to this control, which corresponds to the following formula: RBD binding to coated ACE2 (%) = sample*100/MAX. The half-maximal inhibitory concentration (IC_50_) was calculated using the GraphPad Prism 9 software (GraphPad Software Inc.).

### 4.7 Pseudo-virus neutralization assay

S-pseudo-typed virus particles encoding for luciferase and HEK 293T cells expressing human ACE2 were purchased from VectorBuilder Inc. (Chicago, IL, USA). In this assay, 12.5 × 10^4^ HEK 293T ACE2 cells per well were seeded into 96-well white flat bottom plates. After 7.5 h, S-pseudo-typed virus particles (MOI of 80), 5 µg/ml polybrene, and increasing concentrations of the ACE2-Mb TM or sACE2 were added. As controls, HEK 293T ACE2 cells were cultured solely with 5 µg/ml polybrene in the absence (MIN) or presence (MAX) of S-pseudo-typed virus particles (MOI of 80). Plates were incubated at 37°C and 5% CO_2_ for 65 h. Subsequently, cells were washed with DMEM complete medium, 50 µl One-Glo™ Luciferase Reagent (Promega GmbH) was added, and the bioluminescence signal was measured in the GloMax^®^ Explorer System GM3500 (Promega GmbH). Infectivity was determined according to the following equation: infection (%) = ((sample-MIN)*100)/(MAX-MIN). IC_50_ values were calculated by using GraphPad Prism 9 software (GraphPad Software Inc.).

### 4.8 Plaque reduction neutralization assay

In the PRNT, an early SARS-CoV-2 isolate (GERMANY/GISAID EPI_ISL 406862), the Delta (SARS-CoV-2/CSpecVir25702_4/B.1.617.2 p.1, VS 09.07.2021), and the Omicron (hCoV-19/Netherlands/NH-RIVM-71076/2021) variants acquired from https://www.european-virus-archive.com/evag-news/sars-cov-2-collection were used. Briefly, Vero E6 cells (1.8 × 10^5^ cells/well) were seeded in 12-well plates and incubated overnight. Different concentrations of the ACE2-Mb TM were mixed in equal parts with a virus solution containing 75, 80, or 100 PFU/well of the Delta variant, early isolate, or Omicron variant, respectively, incubated for 1 h at 37°C and subsequently added to the Vero E6 cells, resulting in a total assay volume of 300 µl. The experiment was performed in triplicate, and eight wells were incubated only with the virus solution containing 75, 80, or 100 PFU/well of the Delta variant, early isolate, or Omicron variant, respectively, as a positive control. The virus solution or ACE2-Mb TM/virus solution was incubated with the cells at 37°C for 1 h, followed by supplementation of DMEM medium containing 2% FCS, 0.5% penicillin/streptomycin, and 1.2% Avicel^®^ RC-591 MCC/CMC Sodium (Avicel/DMEM, DuPont, Wilmington, DE, USA). After 2 (Delta variant, early isolate) or 3 days (Omicron variant) at 37°C in Avicel/DMEM, supernatants were removed, the plates were fixed, inactivated using a 6% formaldehyde/PBS solution, and stained with crystal violet to count the number of plaques. The IC_50_ was calculated using a nonlinear regression analysis in the GraphPad Prism 9 software (GraphPad Software Inc.).

### 4.9 Intranasal infection of Syrian hamsters with SARS-CoV-2 and estimation of virus load

All work with live SARS-CoV-2 was performed under BSL-3 conditions at the National Biosafety Laboratory at the National Public Health Centre in Budapest, Hungary (81).

The SARS-CoV-2 Delta VOC (GERMANY/GISAID EPI_ISL 17024327) was obtained through institutional resources. For virus propagation, Vero E6 cells (ATCC^®^ CRL-1586™) were used that were maintained in Gibco^®^ VP-SFM (Thermo Fisher Scientific).

All animal experiments were performed according to the guidelines of the European Communities Council Directive (86/609 EEC) and were approved by the Hungarian National Authority (PE/EA/1456-7/2020). Five male Syrian hamsters (10 weeks old, outbred hamster, RjHan : AURA, Janvier Labs., Le Genest-Saint-Isle, France) were kept under BSL-3 conditions in individually ventilated cages (IsoRat 900N, Techniplast, Italy). Hamsters were fed autoclaved food (VRFI (P), Special Diet Services) *ad libitum*, housed on autoclaved corn bedding (Rehofix, J. Rettenmaier & Söhne GmbH + CO KG, Rosenberg, Germany), and given reverse osmosis-treated water provided in bottles *ad libitum*. All animals were acclimatized at the BSL3 facility prior to the experiments.

A 1.78 × 10^5^ TCID50/ml SARS-CoV-2 Delta VOC (B.1.617.2, EPI_ISL_17024327) in VP-SFM was mixed in equal parts either with PBS (control group) or with the ACE2-Mb TM (2 mg/ml) in PBS (treatment group) right before the inoculation. The hamsters were intranasally inoculated with the respective mixture by pipetting 40 µl volume dropwise into each nostril.

Following infection, the animals were monitored on a daily basis until day 5 post-infection (dpi5). Their body weight was measured daily; in addition, oral/buccal samples were taken on a daily basis in 500 µl DMEM (Lonza Group AG). At dpi5, the animals were humanely euthanized under 3%–5% isoflurane anesthesia with a ketamine/medetomidine mixture (82), and blood samples were taken by venipuncture. In addition, the brain, liver, spleen, lung, kidney, and small intestine were collected. Equal weights of the collected organs were homogenized by MagnaLyser beads (Roche) in DMEM. RNA was isolated using a Qiagen DSP Pathogen Kit (Qiagen) with a QiaSymphony Instrument (Qiagen) according to the manufacturer’s instructions. The virus load was measured by RT-qPCR using a SARS-CoV-2 RT-qPCR kit (PerkinElmer Inc., Waltham, MA, USA) on a Roche LightCycler 480 (Roche) with an automated second derivative evaluation method and automated copy number calculation.

### 4.10 Isolation of human T-cells and generation of UniCAR T-cells

The study was conducted according to the guidelines of the Helsinki Declaration and approved by the local ethics committee of the Medical Faculty Carl Gustav Carus of the TU Dresden (EK138042014). Human T-cells were isolated from buffy coats of healthy donors who gave informed consent (German Red Cross, Dresden, Germany) by Pancoll gradient centrifugation (density: 1.077 g/ml, PAN-Biotech GmbH, Aidenbach, Germany) and negative selection using a Pan T-Cell Isolation Kit (Miltenyi Biotec GmbH) according to the instruction manual. Isolated T-cells were cultured in RPMI complete medium with 50 U/ml human IL-2 (Miltenyi Biotec GmbH) for a few days and either used in cytotoxicity assays (UniMAB system) or transduced with the UniCAR construct as previously described (83). In brief, T-cells were activated with T Cell TransAct™ (Miltenyi Biotec GmbH) according to the manufacturer´s instructions, two to three times transduced with lentiviral particles encoding for the UniCAR construct (MOI of 2, 2 h of spinoculation), and expanded in TEXMACS™ GMP Medium (Miltenyi Biotec GmbH) containing human IL-2, IL-7, and IL-15 (all purchased from Miltenyi Biotec GmbH). Cytokines were removed one day prior to experiments, and transduction efficiency was assessed by measuring the signal of coexpressed EGFP.

### 4.11 Bioluminescence-based cytotoxicity assay and cytokine ELISA

Bioluminescence-based cytotoxicity assays were performed as previously described (48). In brief, T-cells were cocultured with 5 × 10^3^ luciferase-expressing target cells at an effector-to-target cell (*E:T*) ratio of 5:1 without or with 25 nM of the ACE2-Mb TM and/or EM or decreasing concentrations of both Ab components for 24 h. UniCAR T-cells were incubated for 6 h with 5 × 10^3^ luciferase-expressing PC3 cells at an *E*:*T* ratio of 5:1 in the absence or presence of 25 nM or decreasing concentrations of the ACE2-Mb TM.

After incubation, cell-free supernatants were harvested from wells and frozen at −80°C until further analysis. Additionally, 50 µl One-Glo™ Luciferase Reagent (Promega GmbH) per well was added to the cells, the bioluminescence signal was measured using a luminometer (GloMAX^®^ Explorer System GM3500, Promega GmbH or Infinite M200 Pro, Tecan, Männedorf, Switzerland), and the percent specific lysis was determined as previously explained (48). The half-maximal effective concentration (EC_50_) was calculated with GraphPad Prism 9 software (GraphPad Software Inc.).

Moreover, above mentioned supernatants were analyzed by ELISA using a BD OptEIA™ Reagent Set B and a BD OptEIA™ Human IL-2 ELISA Set, BD OptEIA™ Human IFN-γ ELISA Set, or BD OptEIA™ Human TNF ELISA Set (all purchased from BD Biosciences) according to the manufacturer’s instructions. To quantify the cytokines, a standard curve ranging from 7.8 to 500 pg/ml was included. Values below the detection level were set to 0.

### 4.12 Statistical analysis

Statistical analysis was performed using GraphPad Prism 9 software (GraphPad Software Inc.), as indicated in the respective figure legend. *p*-values less than 0.05 were considered statistically significant.

## Data availability statement

The original contributions presented in the study are included in the article/**Supplementary Material**. Further inquiries can be directed to the corresponding author.

## Ethics statement

The studies involving human participants were reviewed and approved by local ethics committee of the Medical Faculty Carl Gustav Carus of the TU Dresden (EK138042014). The patients/participants provided their written informed consent to participate in this study. The animal study was reviewed and approved by Hungarian National Authority (PE/EA/1456-7/2020).

## Author contributions

Conceptualization: MB, AF, and AKe. Methodology and investigation: LD, AKe, AM-S, AKü, ML, RB, KG, TB, DM, BP, PH, LF, ZK, and KS. Data curation and formal analysis: LD, AKe, AM-S, AKü, ML, RB, DM, BP, PH, LF, ZK, and KS. Design of the ACE2-Mb TM: AF. Cloning of the ACE2-Mb TM: AKe. Design and cloning of the Mb backbone-containing vector: CA. Cloning of the vector pHIT-basisDHFR_Immunovid19 and establishment of the ACE2-Mb TM-expressing CHO-HITB7 cell line: HZ, MH, and AN. Resources: MB, AF, BS, JD, LB, CD, CN, NM, and LR. Visualization: LD and AKe. Validation: LD, AKe, RB, AF, and MB. Writing —original draft: AKe, LD, MB, RB, MH, ML, and AM-S. Writing —review and editing: AKe, LD, MB, RB, CA, AF, KG, TB, HZ, MH, AN, ML, AM-S, JD, AKü, DM, BP, PH, LF, ZK, KS, BS, LB, CD, CN, NM, and LR. Supervision and project administration: MB, AF, and AKe. Funding acquisition: MB. All authors have read and agreed to the published version of the manuscript.
