# Supplementary material for: A novel ACE2 decoy for both neutralization of SARS-CoV-2 variants and killing of infected cells
Source: Front Immunol. 2023 Jun 13;14:1204543. doi: 10.3389/fimmu.2023.1204543 (PMC10293748; doi:10.3389/fimmu.2023.1204543)
Supplement: Supplementary file 1 [file DataSheet_1.pdf]

Supplementary Material

A novel ACE2 decoy for both neutralization of SARS-CoV-2 variants and killing of infected cells

Alexandra Kegler[1†], Laura Drewitz[1†], Claudia Arndt[1,2], Cansu Daglar[1], Liliana Rodrigues Loureiro[1], Nicola Mitwasi[1], Christin Neuber[1], Karla Elizabeth González Soto[1], Tabea Bartsch[1], Larysa Baraban[1], Holger Ziehr[3], Markus Heine[3], Annabel Nieter[3], Andres Moreira-Soto[4], Arne Kühne[4], Jan Felix Drexler[4], Barbara Seliger[5,6], Markus Laube[7], Domokos Máthé[8,9,10], Bernadett Pályi[11], Polett Hajdrik[8], László Forgách[12], Zoltán Kis[11], Krisztián Szigeti[8], Ralf Bergmann[1,8], Anja Feldmann[1,13,14], Michael Bachmann[1,13,14]*

*** Correspondence: Michael Bachmann: m.bachmann@hzdr.de**

1 Supplementary Figures and Tables

1.1 Supplementary Figures

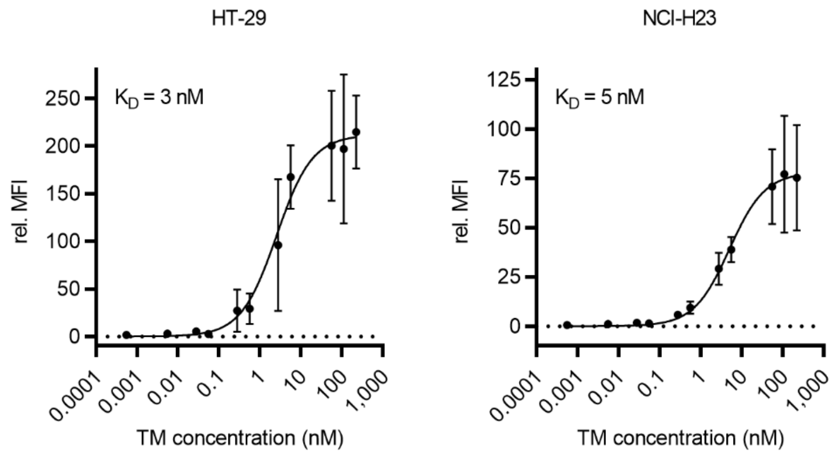

Supplementary Figure 1: Binding of the ACE2-Mb TM to SARS-CoV-2 RBD⁺ cell lines of colorectal (HT-29) or lung (NCI-H23) origin. Cells were incubated with increasing concentrations of ACE2-Mb TM. Binding was subsequently detected using as primary Ab the anti-La mAb (5B9) and a fluorescently-labeled, secondary goat anti-mouse IgG (minimal-x-reactivity) Ab and evaluated via flow cytometry. Binding affinity curves were generated by plotting the relative median fluorescence intensity (rel. MFI) against TM concentration. Summarized data as mean ± SD and respective K_D values are shown (n=3).

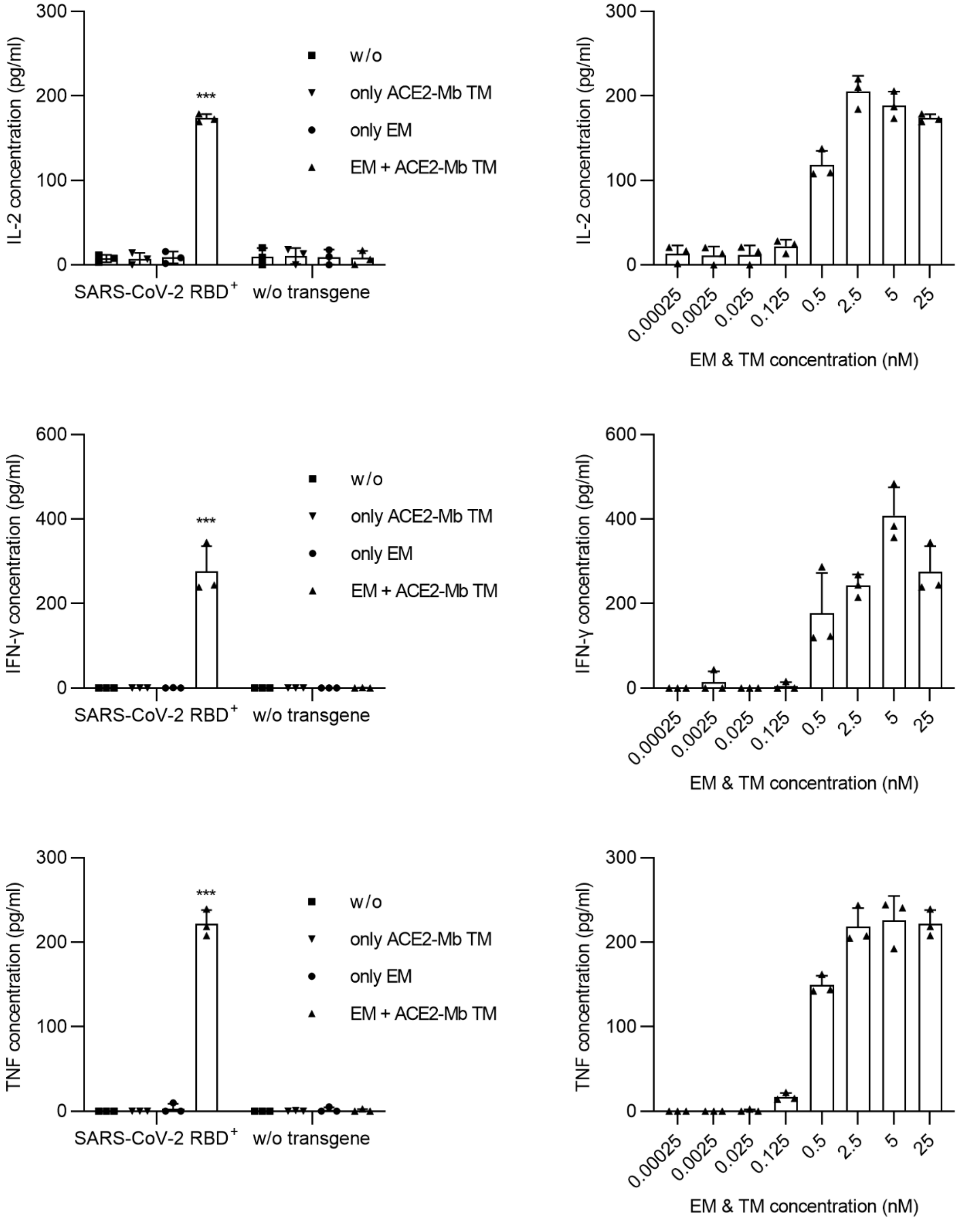

Supplementary Figure 2: Recruitment of T-cells via the UniMAB system results in specific cytokine release in the presence of the ACE2-Mb TM. PC3 cells with (SARS-CoV-2 RBD^{+}, left and right diagrams) or without (w/o transgene, left diagrams) SARS-CoV-2 RBD surface expression were co-cultured with T-cells at an *E:T* ratio of 5:1. Co-cultivation was performed either in the absence (w/o) or presence of 25 nM (left diagrams) or decreasing concentrations (right diagrams) of indicated Ab components. After 24 h, supernatants were harvested and analyzed by ELISA. Data of three

individual donors are summarized as mean ± SD. Statistical analysis was performed by using two-way ANOVA with Tukey's multiple comparisons test (*** $p < 0.001$ with respect to w/o, only ACE2-Mb TM and only EM controls).

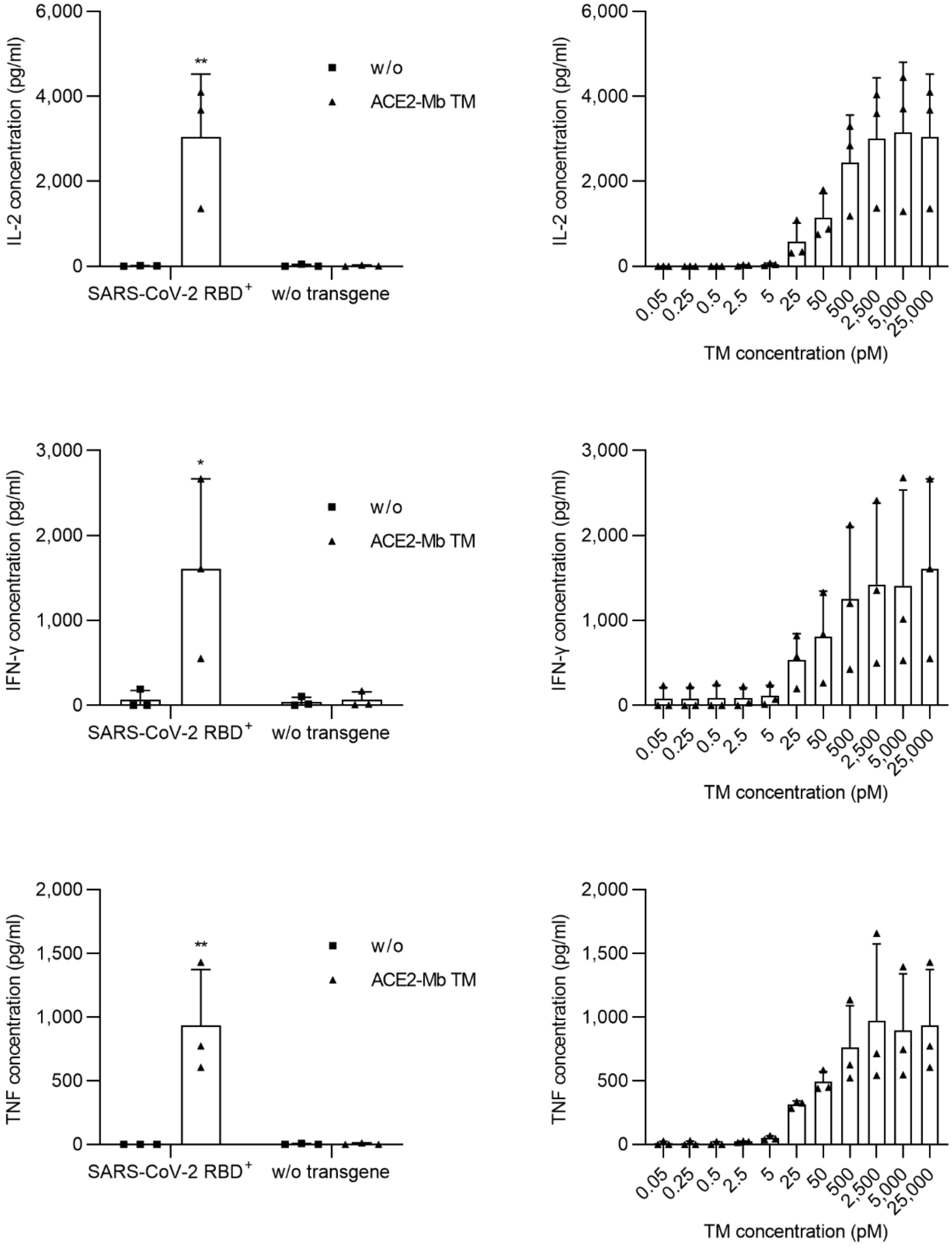

Supplementary Figure 3: Recruitment of T-cells via the UniCAR system results in specific cytokine release in the presence of the ACE2-Mb TM. SARS-CoV-2 RBD positive (SARS-CoV-2 RBD$^+$, left and right diagrams) or negative (w/o transgene, left diagrams) PC3 cells were incubated with UniCAR T-cells at an *E:T* ratio of 5:1 either without (w/o), with 25 nM (left diagrams) or decreasing (right diagrams) TM concentrations for 6 h. Afterwards, supernatants were harvested and cytokine amounts were measured by ELISA. Data of three individual donors are shown as mean ± SD.

Statistical analysis was performed by using two-way ANOVA with Šidák's multiple comparisons test (* $p < 0.05$ and ** $p < 0.01$ with respect to w/o controls).

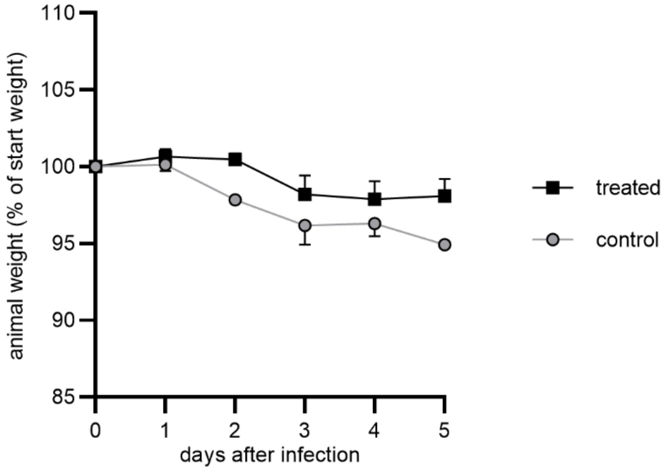

Supplementary Figure 4: Comparison of the body weight of SARS-CoV-2 infected golden hamsters treated with the ACE2-Mb TM versus control animals. Golden hamsters were infected by intranasal application of a SARS-CoV-2 Delta VOC containing solution, which was mixed before in equal parts either with the ACE2-Mb TM in PBS (treated, black) or only PBS (control, grey). The body weight of the animals was measured for five days after infection and normalized to the starting body weight. Summarized data are shown as mean plus (treated) or minus (control) SEM.

1.2 Supplementary Tables

Supplementary Table 1. Results of SPR measurements. Results are given as mean ± SD of two independent experiments each analyzed in triplicate (n = 6).

	ka (1/Ms)	kd (1/s)	K_D (nM)
SARS-CoV-2 RBD_{WT}	$1.78 \pm 0.79 \times 10^5$	$3.64 \pm 0.21 \times 10^{-4}$	2.05 ± 0.05
SARS-CoV-2 RBD_{Delta}	$21.81 \pm 11.80 \times 10^5$	$9.87 \pm 2.02 \times 10^{-4}$	0.61 ± 0.36
SARS-CoV-2 $RBD_{Omicron}$	$78.63 \pm 8.58 \times 10^5$	$42.49 \pm 10.17 \times 10^{-4}$	0.54 ± 0.10
